# Demirjian Four-Teeth Age Estimation in Thai Children and Adolescents: A Population-Specific Radiographic Validation Study

**DOI:** 10.3390/jpm16080408

**Published:** 2026-07-30

**Authors:** Pennipat Nabheerong, Phuwadon Duangto, Suttiwat Jeamtrakool, Chairat Charoemratrote, Pornpat Theerasopon

**Affiliations:** 1 Department of Radiology, School of Medicine, University of Phayao, Phayao 56000, Thailand; pennipat.na@up.ac.th (P.N.); suttiwat.je@up.ac.th (S.J.); 2 Division of Anatomy, School of Medical Sciences, University of Phayao, Phayao 56000, Thailand; pete_anatomy@hotmail.com; 3 Department of Preventive Dentistry, Faculty of Dentistry, Prince of Songkla University, Songkhla 90112, Thailand; metalbracket@outlook.co.th; 4 Department of Orthodontics, School of Dentistry, University of Phayao, Phayao 56000, Thailand

**Keywords:** adolescent, child, dental age estimation, forensic dentistry, population-specific calibration, radiography, panoramic

## Abstract

**Background/Objectives**: Dental age estimation is widely used in clinical and forensic practice. The Demirjian four-teeth methods were developed to simplify age assessment; however, their accuracy varies among populations and requires validation before use in specific ethnic groups. This study aimed to evaluate and compare the accuracy of Demirjian’s four-teeth methods I and II for dental age estimation in Southern Thai children and adolescents aged 7–15 years. **Methods**: This retrospective cross-sectional study included 360 digital panoramic radiographs (180 males and 180 females) from Southern Thai individuals aged 7–15 years. Dental age was estimated using Demirjian’s four-teeth method I (teeth 34, 35, 36, and 37) and method II (teeth 31, 34, 35, and 37). Estimated dental ages were compared with chronological ages using the Wilcoxon signed-rank test. Accuracy was assessed by mean error (ME), mean absolute error (MAE), and root mean square error (RMSE). Intra- and inter-observer reliability were evaluated using weighted kappa statistics. **Results**: Intra- and inter-observer agreement were excellent, ranging from 0.957 to 1.000 and 0.895 to 0.956, respectively. The mean chronological age was 11.53 ± 2.55 years. Both methods overestimated chronological age in males and females. ME ranged from 0.54 to 0.75 years, while MAE ranged from 0.97 to 1.01 years. Method II produced slightly lower estimation errors than method I. Significant differences between dental and chronological ages were found for both methods in both sexes (*p* < 0.05). **Conclusions**: Both methods overestimated chronological age by approximately one year in Southern Thai children and adolescents. Method II showed marginally lower errors; however, population-specific calibration may improve age estimation in Thai populations. These findings support the need for population-specific dental age estimation strategies, contributing to more personalized and accurate age estimation for Thai children in both clinical and forensic settings.

## 1. Introduction

Age estimation plays an essential role in both clinical practice and forensic sciences, particularly during childhood and adolescence when growth and developmental changes occur rapidly. In clinical settings, age estimation assists in evaluating growth and maturation status, treatment planning in pediatric dentistry and orthodontics, and the assessment of developmental disorders. In forensic contexts, age estimation contributes to personal identification in living and deceased individuals, including undocumented migrants, unaccompanied minors, disaster victims, and individuals lacking reliable birth records [1]. In forensic practice, an estimation error within 0.5–1 year is generally considered acceptable, given its implications for legal and humanitarian decisions [2,3].

Dental age estimation can be performed using either two-dimensional (2D) or three-dimensional (3D) [4] radiographic techniques. Although cone-beam computed tomography (CBCT) provides detailed anatomical information, panoramic radiography remains the most widely used imaging modality for dental age estimation because it is routinely available in dental practice, cost-effective, associated with relatively low radiation exposure, and suitable for large-scale retrospective studies [5]. Consequently, most population-based studies evaluating dental age estimation methods continue to rely on panoramic radiographs.

Several biological indicators have been proposed for age estimation, including dental development [6], skeletal maturation [7], and sexual maturation. Among these indicators, dental development is generally regarded as one of the most reliable because tooth formation is predominantly controlled by genetics and is less influenced by environmental, nutritional, and socioeconomic factors than skeletal growth [8]. Previous studies have demonstrated that dental development exhibits a strong relationship with chronological age and provides superior reproducibility compared with many other biological age indicators [9].

Among the various dental age estimation methods currently available, the method developed by Demirjian, Goldstein, and Tanner remains one of the most extensively investigated and internationally accepted approaches [6]. The Demirjian method evaluates the developmental stages of seven left mandibular permanent teeth using an eight-stage classification system ranging from stage A to stage H. One of the major strengths of this method is the use of clearly defined developmental criteria accompanied by illustrative reference diagrams, which facilitate examiner calibration and contribute to high intra- and inter-observer reliability [10]. As a result, it has become one of the most frequently applied techniques in pediatric dentistry, forensic odontology, and anthropological research. Despite its widespread acceptance, Demirjian’s seven-tooth method has several practical limitations. The method requires all seven mandibular permanent teeth to be present and clearly visible on radiographs, making its application difficult in individuals with missing teeth or developmental anomalies, or in cases with poor radiographic quality. To address these limitations, Demirjian and Goldstein subsequently introduced alternative approaches based on four mandibular permanent teeth [11]. Two four-teeth systems were proposed: method I, which evaluates the first premolar (tooth 34), second premolar (tooth 35), first molar (tooth 36), and second molar (tooth 37), and method II, which evaluates the central incisor (tooth 31), first premolar (tooth 34), second premolar (tooth 35), and second molar (tooth 37) [11]. These simplified approaches reduce the number of teeth required for assessment while maintaining acceptable levels of accuracy and reproducibility.

The applicability of Demirjian’s four-teeth methods has been investigated in several populations worldwide. Studies conducted in Australia, Turkey, Tunisia, Kosovo, Kenya, India, Saudi Arabia, and Thailand have demonstrated that the performance of these methods varies considerably among populations [12,13,14,15,16,17,18,19]. Flood et al. reported that the four-teeth method provided the lowest mean age difference among South Australian children [13]. Similarly, Yassin demonstrated that the four-teeth approach was the most accurate method among Saudi Arabian males, while the revised seven-teeth system performed better in females [18]. Collectively, these findings indicate that the accuracy of Demirjian-based age estimation methods is population-dependent and should be validated before clinical or forensic application in a specific population. Most studies reported overestimation or underestimation of chronological age, suggesting that dental maturation patterns differ according to ethnic, genetic, environmental, and socioeconomic factors [9,12,13,14,15,16,17,18,19].

The observed variability among populations highlights the importance of population-specific validation studies. Differences in genetic background, ethnicity, environmental influences, nutritional status, and socioeconomic conditions may affect the timing and progression of dental maturation, thereby influencing the accuracy of age estimation methods derived from reference populations [9]. Consequently, direct application of standards developed in one population to another may result in systematic estimation errors and reduced forensic reliability.

For the Thai population, evidence regarding the applicability of Demirjian and Goldstein’s four-teeth methods remains limited. Duangto et al. developed population-specific equations based on four permanent mandibular teeth in Northern Thai children and adolescents and demonstrated improved accuracy compared with Demirjian’s methods [19]. However, information regarding the performance of Demirjian’s four-teeth methods in other regions of Thailand remains scarce. Given the potential influence of regional demographic, environmental, and socioeconomic factors on dental maturation, further validation studies in different Thai populations are warranted. Such investigations are essential to determine whether the original standards can be reliably applied across Thai populations or whether regional calibration may be required. Accordingly, this study is the first external validation of Demirjian’s four-teeth methods in Southern Thai children and adolescents.

We hypothesized that both Demirjian’s four-teeth methods would systematically overestimate chronological age in Southern Thai children and adolescents. Therefore, the present study aimed to evaluate and compare the accuracy of Demirjian’s four-teeth methods I and II in Southern Thai children and adolescents aged 7–15 years using digital panoramic radiographs. The findings of this study will provide additional evidence regarding the applicability of these methods for age estimation in Thai children and adolescents and may contribute to the development of more reliable forensic and clinical age estimation standards for the Thai population.

## 2. Materials and Methods

The study protocol was approved by the Human Research Ethics Committee, Faculty of Dentistry, Prince of Songkla University, Songkhla, Thailand (No. EC6810–050). The sample size was calculated using G*Power software (version 3.1.9.7) based on a matched-pairs *t*-test, assuming an effect size of 0.20, a statistical power of 95%, and a significance level of 5% (α = 0.05). An effect size of 0.20 was selected because the expected difference between paired measurements was small. Therefore, the study was designed to provide adequate statistical power to detect small but meaningful differences. The minimum required sample size was determined to be 327 participants. This study was conducted using a sample of 360 digital panoramic radiographs obtained through simple random sampling from Thai individuals aged 7–15 years, comprising 180 males and 180 females (Table 1). Age was recorded in years according to the metric system, with two decimal places representing months. Participants were identified as belonging to the Thai population based on Thai first and last names. The radiographs were acquired using the GXDP-700 PANOREX + cone-beam machine (Gendex Dental Systems, Hatfield, PA, USA). All radiographs were collected from the Dental Hospital of the Faculty of Dentistry, Prince of Songkla University, Songkhla, Thailand (between 2015 and 2025). All panoramic radiographs were obtained using a single panoramic imaging device with standardized exposure protocols maintained throughout the study period. Exclusion criteria included non-Thai individuals, unclear radiographs, abnormalities in tooth development, and the presence of jaw cysts or tumors. Additionally, this study adhered to the STrengthening the Reporting of OBservational studies in Epidemiology (STROBE) checklist for cross-sectional studies (Supplementary Table S1).

The performance of Demirjian’s four-teeth methods I and II was evaluated. Two sets of four teeth were analyzed. Method I consisted of teeth 34, 35, 36, and 37, whereas method II included teeth 31, 34, 35, and 37. The developmental stage of each tooth was assessed and classified into one of eight stages, from A through H, according to the method proposed by Demirjian et al. [11]. Estimated dental ages obtained from each method were compared with the chronological ages of 360 participants. Descriptive statistics, including minimum, maximum, mean, and standard deviation, were calculated for chronological age and dental age. Accuracy was assessed by calculating the difference between estimated dental age and chronological age. Positive values represented age overestimation, whereas negative values indicated age underestimation. Mean error (ME), mean absolute error (MAE), and root mean square error (RMSE) were calculated to assess the direction, magnitude, and variability of estimation errors, respectively. The Wilcoxon signed-rank test was used to compare estimated dental ages with chronological ages, as the normality test demonstrated that the paired differences did not satisfy the assumption of normality.

Before image assessment, both examiners underwent calibration to ensure consistent application of the Demirjian method. Subsequently, two calibrated examiners independently assessed tooth developmental stages on 40 randomly selected digital panoramic radiographs according to the method of Demirjian et al. Each examiner repeated the assessment of the same radiographs after a one-month interval. Intra- and inter-observer agreement were analyzed using weighted kappa statistics. All statistical analyses were performed using SPSS Statistics version 27 (SPSS for Windows, version 27; Chicago, IL, USA).

## 3. Results

Weighted kappa values showed almost perfect reproducibility of 0.957 to 1.000 for intra-observer agreement and 0.895 to 0.956 for inter-observer agreement (Table 2).

Among a total of 360 participants (180 males and 180 females), the mean chronological age was 11.53 ± 2.55 years. The mean estimated dental age was 12.22 ± 2.67 years for method I and 12.14 ± 2.78 years for method II, as reported in Table 3. When analyzed separately by sex, significant differences were observed in both method I and method II in both sexes. Males had a mean chronological age of 11.53 ± 2.54 years, while the estimated dental age from method I was 12.28 ± 2.67 years, with MAE of 0.97 years, and the estimated dental age from method II was 12.22 ± 2.82 years, with MAE of 0.99 years. The mean chronological age in females was 11.53 ± 2.56 years, while the estimated dental age from method I was 12.17 ± 2.68 years, with MAE of 1.01 years, and the estimated dental age from method II was 12.07 ± 2.75 years, with MAE of 1.01 years (Table 4). Overall, the Demirjian four-teeth methods tended to overestimate age in both males and females by approximately one year.

In males, the ME was 0.75 years for Demirjian four-teeth method I and 0.69 years for method II, with statistically significant difference (*p* < 0.05). In females, the ME was 0.65 years for method I and 0.54 years for method II, with statistically significant difference (*p* < 0.05). The overall MAE between the estimated dental age and chronological age in both methods was 0.97–0.99 years in males and 1.01 years in females (Table 3). When analyzed separately, the MAE was 0.97 years for method I and 1.01 years for method II in males, and 0.99 years for method I and 1.01 years for method II in females. These findings demonstrate that both four-teeth methods provide reliable and consistent age estimation results for both males and females, with an error range of approximately one year. The RMSE showed different values between the estimated dental age and chronological age in both methods, ranging from 1.24 to 1.28 years in males and from 1.34 to 1.38 years in females.

## 4. Discussion

The present study evaluated the applicability of the Demirjian four-teeth methods for dental age estimation in Southern Thai children and adolescents aged 7–15 years. The results demonstrated that both four-teeth methods overestimated chronological age in both males and females. The ME ranged from 0.54 to 0.75 years, whereas the MAE ranged from 0.97 to 1.01 years. These findings indicate that although the methods provide a practical approach for age estimation, they tend to overestimate age in Southern Thai children and adolescents.

The tendency toward age overestimation observed in the present study is consistent with previous reports evaluating the Demirjian method in populations other than the original French-Canadian reference sample [11]. Dental maturation patterns vary among populations and may be influenced by genetic background, ethnicity, environmental conditions, socioeconomic status, and nutritional factors. Consequently, direct application of the original Demirjian standards may lead to overestimation or underestimation of chronological age in different populations [9,20].

In 2016, Mbithe et al. evaluated the applicability of Demirjian’s four-teeth method for dental age estimation among Kenyan children aged 6–12 years. The findings demonstrated that the method slightly overestimated chronological age by an average of 0.53 years, with a greater overestimation observed in females than in males. Despite this discrepancy, the method showed acceptable accuracy and was considered reasonably applicable for age estimation in the studied Kenyan population [15]. In 2017, Mishra et al. compared Demirjian’s four-teeth method and an alternate four-teeth method for dental age estimation among children and adolescents in the Varanasi region of India. Both methods demonstrated a strong correlation between estimated dental age and chronological age; however, their accuracy differed by sex. The Demirjian four-teeth method was more accurate in boys, while the alternate four-teeth method performed better in girls. Overall, both methods were considered applicable for the studied population, although population-specific variations in dental maturation were observed [16].

In a sample of 799 Turkish children, Akkaya et al. evaluated several Demirjian approaches, including the four-teeth methods, and compared them with the Willems method. They reported that the Demirjian methods generally tended to overestimate chronological age, whereas the Willems method produced lower mean errors in the Turkish population [12]. Similarly, Kelmendi et al. compared four methods, the Demirjian, Chaillet, and Willems methods, in a study of 1022 Kosovar children. They found that the Willems method demonstrated superior overall accuracy, while the Demirjian four-teeth methods still showed tendencies toward overestimation or underestimation depending on age group and sex [14]. Furthermore, Nemsi et al., working with a Tunisian pediatric cohort, also reported that Demirjian’s four-teeth method yielded greater mean errors compared with Willems’ method. Collectively, these findings indicate that applying the original methods to different populations often requires calibration or the development of new population-specific equations [17].

When compared with previous Thai studies, the present investigation demonstrated greater age overestimation. Duangto et al. developed population-specific equations based on four mandibular permanent teeth and reported substantially lower mean errors in Northern Thai children and adolescents [19]. The discrepancy between the present findings and those reported by Duangto et al. may reflect regional differences in dental maturation patterns among Thai children. Variations in genetic background, environmental influences, nutritional status, socioeconomic conditions, and secular trends in growth and development may contribute to these differences. However, these potential explanations remain speculative, as genetic, nutritional, socioeconomic, and environmental factors were not directly assessed in the present study. Additional multicenter studies involving different geographic regions of Thailand are therefore warranted to further investigate these potential sources of variation.

Demirjian’s four-teeth method II demonstrated slightly lower mean errors than method I in both males and females. Although the improvement in accuracy was modest, the findings suggest that tooth selection may influence age estimation. Nevertheless, both methods showed a similar tendency toward age overestimation. These findings are consistent with previous reports indicating that population-specific calibration may improve the accuracy of Demirjian age estimation methods [9,19].

The present study was designed as an external validation of the Demirjian four-teeth methods rather than the development of a new age estimation model. Validation studies are essential because methods that perform well in one population may not demonstrate equivalent performance in another. The findings therefore provide additional evidence regarding the applicability of the Demirjian four-teeth methods in Southern Thailand and contribute to the growing body of literature supporting population-specific evaluation of dental age estimation techniques [12,14,17].

The findings of this study have important implications for both forensic and clinical practice. From a forensic perspective, an average overestimation of approximately one year may be clinically and legally relevant, particularly for individuals whose chronological age is close to a legally defined age threshold. Such systematic overestimation could result in age misclassification and potentially influence legal or administrative decisions that depend on whether an individual is considered to be above or below a specific age threshold. Therefore, dental age estimates should be interpreted cautiously and considered alongside other available clinical and forensic evidence, particularly in borderline cases. In clinical practice, the methods may still provide useful information regarding developmental status from conventional routine dental radiographs for age estimation; however, clinicians should be aware of the potential for age overestimation in Southern Thai children and adolescents.

Several alternative approaches have been proposed for dental age estimation, including the methods of Willems [21] and the London Atlas [5,22,23]. Previous studies have reported that these methods may provide improved accuracy in certain populations. Future studies should directly compare these approaches with the Demirjian four-teeth methods and evaluate whether population-specific equations can improve age estimation accuracy in Thai children and adolescents [5,12,13,14,22].

Several limitations should be acknowledged. First, the retrospective design may be subject to inherent biases and limitations in the availability of clinical information. As the study was based on hospital-derived radiographs, selection bias may also be present, and the study population may not fully represent the general population. Therefore, the generalizability of our findings should be interpreted with caution. Second, only individuals aged 7–15 years were included; therefore, the results may not be applicable to younger children or older adolescents. Third, the study evaluated only the Demirjian four-teeth methods and did not directly compare their performance with other commonly used age estimation techniques, such as the Willems method or the London Atlas method. Finally, the Demirjian standards were applied without population-specific recalibration, which may have contributed to the observed overestimation of chronological age. Future multicenter studies should develop population-specific calibration models using larger and more representative Thai samples and subsequently validate these models in independent cohorts to improve the accuracy and generalizability of age estimation.

## 5. Conclusions

Both Demirjian four-teeth methods demonstrated a tendency to overestimate chronological age in Southern Thai children and adolescents. The ME ranged from 0.54 to 0.75 years, while the MAE was approximately one year. Demirjian four-teeth method II showed marginally lower errors than method I; however, both methods exhibited similar patterns of age overestimation. These findings suggest that population-specific calibration of regional reference standards may improve age estimation accuracy in Thai children and adolescents.

## Figures and Tables

**Table 1:** Distribution of samples by age and sex.

Age Group	Male	Female	Overall
7	20	20	40
8	20	20	40
9	20	20	40
10	20	20	40
11	20	20	40
12	20	20	40
13	20	20	40
14	20	20	40
15	20	20	40
Total	180	180	360

**Table 2:** Weighted kappa values of intra- and inter-observer agreement of dental development using the Demirjian method [6].

Teeth	Intra-Observer Agreement	Inter-Observer Agreement
31	1.000	0.956
34	1.000	0.924
35	0.957	0.895
36	1.000	0.948
37	0.984	0.952

**Table 3:** Descriptive statistics of the chronological age (CA) and estimated dental age (DA).

Sex	Variable	n	Minimum	Maximum	Mean	Standard Deviation
Male	CA	180	7.23	15.99	11.53	2.54
	DA from method I	180	7.00	16.00	12.28	2.67
	DA from method II	180	7.00	16.00	12.22	2.82
Female	CA	180	7.03	15.95	11.53	2.56
	DA from method I	180	6.70	15.90	12.17	2.68
	DA from method II	180	6.70	16.00	12.07	2.75
Overall	CA	360	7.03	15.99	11.53	2.55
	DA from method I	360	6.70	16.00	12.22	2.67
	DA from method II	360	6.70	16.00	12.14	2.78

n = participants; method I: Demirjian four-teeth method I using teeth 34, 35, 36, 37; method II: Demirjian four-teeth method II using teeth 31, 34, 35, 37.

**Table 4:** Mean error (ME), mean absolute error (MAE), and root mean square error (RMSE) values using the Demirjian four-teeth methods in both sexes.

Sex	Method	ME (Years) (CI) (*p*-Value)	MAE (Years)	RMSE (Years)
Male (n = 180)	Method I	0.75 * (0.60–0.89) (*p* < 0.001)	0.97	1.24
	Method II	0.69 * (0.53–0.85) (*p* < 0.001)	0.99	1.28
Female (n = 180)	Method I	0.65 * (0.47–0.82) (*p* < 0.001)	1.01	1.34
	Method II	0.54 * (0.35–0.73) (*p* < 0.001)	1.01	1.38

n = participants; Method I: Demirjian four-teeth method I using teeth 34, 35, 36, 37; Method II: Demirjian four-teeth method II using teeth 31, 34, 35, 37; CI = confidence interval. * Statistically significant difference using Wilcoxon signed-rank test (*p* < 0.05).

## Data Availability

Data supporting the findings of this study are available from the corresponding author upon reasonable request.

## Supplementary Materials

The following supporting information can be downloaded at: https://www.mdpi.com/article/10.3390/jpm16080408/s1, Table S1: STROBE Statement—checklist of items that should be included in reports of observational studies.

## Abbreviations

The following abbreviations are used in this manuscript:

MAE	Mean absolute error
ME	Mean error
RMSE	Root mean square error
Tooth 31	Left mandibular central incisor
Tooth 34	Left mandibular first premolar
Tooth 35	Left mandibular second premolar
Tooth 36	Left mandibular first molar
Tooth 37	Left mandibular second molar
